# Supplementary material for: The Systems Biology Research Tool: evolvable open-source software
Source: BMC Syst Biol. 2008 Jun 29;2:55. doi: 10.1186/1752-0509-2-55 (PMC2446383; doi:10.1186/1752-0509-2-55)
Supplement: Additional file 1 — SBRT Archive. An archive of the current version of the Systems Biology Research Tool. [file 1752-0509-2-55-S1.zip › sbrt-1.4.0/doc/developers_guide/api/sbrt/kernel/math/LinearComb.html]

LinearComb


|  |  |  |
| --- | --- | --- |
| **Overview** \| **Package** \| **Class** \| **Use** \| **Tree** \| **Deprecated** \| **Index** \| **Help** | |  |
| **PREV CLASS**   **NEXT CLASS** | **FRAMES**    **NO FRAMES**     **All Classes** |  |
| SUMMARY: NESTED \| FIELD \| CONSTR \| METHOD | DETAIL: FIELD \| CONSTR \| METHOD |  |


---


## sbrt.kernel.math Interface LinearComb<V>

**Type Parameters:**: `V` - the variable type.

**All Superinterfaces:**: java.lang.Iterable<LinearComb.Term<V>>, MathExpr<V>, Polynomial<V>

**All Known Implementing Classes:**: AbstractLinearComb, HashLinearComb

---

``` public interface LinearComb<V> extends Polynomial<V>, java.lang.Iterable<LinearComb.Term<V>> ```

This interface is used to represent linear combinations. A linear
combination is a type of polynomial.

**Author:**
:   This interface was written and documented by
    Jeremiah Wright while in the Wagner lab.

---

| **Nested Class Summary** | |
| --- | --- |
| `static interface` | `LinearComb.Term<V>`             This interface is used to represent the terms of linear combinations. |


| **Method Summary** | |
| --- | --- |
| `LinearComb<V>` | `add(double constant)`             Adds, in the mathematical sense, the provided constant to this linear combination. |
| `LinearComb<V>` | `add(LinearComb<? extends V> linComb)`             Adds, in the mathematical sense, the provided linear combination to this linear combination. |
| `boolean` | `equals(java.lang.Object obj)`             Indicates if the provided object is equal to this linear combination. |
| `double` | `getCoefficient(java.lang.Object variable)`             Returns the coefficient of the term containing the provided variable. |
| `LinearComb.Term<V>[]` | `getTermArray()`             Returns the terms of this linear combination as an array. |
| `java.util.List<LinearComb.Term<V>>` | `getTermList()`             Returns the terms of this linear combination as list. |
| `double` | `getZeroCutoff()`             Returns the current zero cutoff. |
| `int` | `hashCode()`             Returns a content-based hash code for this linear combination. |
| `java.util.Iterator<LinearComb.Term<V>>` | `iterator()`             Returns an iterator over the terms of this linear combination. |
| `LinearComb<V>` | `multiply(double constant)`             Multiplies this linear combination by the provided constant. |
| `LinearComb<V>` | `plugIn(java.util.Map<?,? extends LinearComb<? extends V>> linearCombs)`             Plugs in the provided linear combinations to produce a new linear combination. |
| `LinearComb<V>` | `plugIn(java.util.Map<?,java.lang.Double> numbers, java.util.Map<?,? extends LinearComb<? extends V>> linearCombs)`             Plugs in the provided numerical values and linear combinations to produce a new linear combination. |
| `LinearComb<V>` | `plugIn(java.lang.Object variable, double constant)`             Assigns the value of the provided variable to the provided constant. |
| `LinearComb<V>` | `plugIn(java.lang.Object variable, LinearComb<? extends V> expr)`             Assigns the value of the provided variable to the provided linear combination. |
| `LinearComb<V>` | `plugInNumbers(java.util.Map<?,java.lang.Double> numbers)`             Plugs in the provided numerical values to produce a new linear combination. |
| `LinearComb<V>` | `solveFor(java.lang.Object variable, double constant)`             Equates this linear combination to the provided constant and solves for the provided variable. |
| `java.lang.String` | `toString()`             Returns an appropriate string representation of this linear combination. |

**Methods inherited from interface sbrt.kernel.math.Polynomial**

`getConstant, terms`

**Methods inherited from interface sbrt.kernel.math.MathExpr**

`getVariables, hasAllVariables, hasVariable, hasVariables, variables`

**Method Detail**

### getZeroCutoff

```
double getZeroCutoff()
```

:   Returns the current zero cutoff.

    The zero cutoff is a value used to decide if coefficients are close
    enough to zero to be considered equal to zero. In other words, the
    absolute value of the coefficient of every term of this linear
    combination will always be greater than the zero cutoff.

    :   **Returns:**: the zero cutoff.

---


### getCoefficient

```
double getCoefficient(java.lang.Object variable)
```

:   Returns the coefficient of the term containing the provided variable.

    :   **Parameters:**: `variable` - the variable whose corresponding coefficient is to be returned. **Returns:**: the coefficient of the term containing the provided variable.

---


### add

```
LinearComb<V> add(LinearComb<? extends V> linComb)
```

:   Adds, in the mathematical sense, the provided linear combination to this
    linear combination.

    :   **Parameters:**: `linComb` - the linear combination with which to add. **Returns:**: the result of adding this linear combination with the one provided.

---


### add

```
LinearComb<V> add(double constant)
```

:   Adds, in the mathematical sense, the provided constant to this
    linear combination.

    :   **Parameters:**: `constant` - the constant with which to add. **Returns:**: the result of adding this linear combination with the provided constant.

---


### multiply

```
LinearComb<V> multiply(double constant)
```

:   Multiplies this linear combination by the provided constant.

    :   **Parameters:**: `constant` - the constant with which to multiply this linear combination. **Returns:**: the result of multiplying this linear combination by the provided constant.

---


### solveFor

```
LinearComb<V> solveFor(java.lang.Object variable,
                       double constant)
```

:   Equates this linear combination to the provided constant and solves for
    the provided variable.

    :   **Parameters:**: `variable` - the variable in this linear combination for which to solve.: `constant` - the constant with which this linear combination is equated. **Returns:**: a new linear combination representing the value of the provided variable.

---


### plugIn

```
LinearComb<V> plugIn(java.lang.Object variable,
                     LinearComb<? extends V> expr)
```

:   Assigns the value of the provided variable to the provided linear
    combination.

    :   **Parameters:**: `variable` - the variable in this linear combination whose value will be assigned the provided linear combination.: `expr` - the linear combination to be "plugged in". **Returns:**: the result of "plugging in" the provided linear combination for the provided variable.

---


### plugIn

```
LinearComb<V> plugIn(java.lang.Object variable,
                     double constant)
```

:   Assigns the value of the provided variable to the provided constant.

    :   **Parameters:**: `variable` - the variable in this linear combination whose value will be assigned the provided constant.: `constant` - the value to be "plugged in". **Returns:**: the result of "plugging in" the provided constant for the provided variable.

---


### plugInNumbers

```
LinearComb<V> plugInNumbers(java.util.Map<?,java.lang.Double> numbers)
```

:   Plugs in the provided numerical values to produce a new
    linear combination.

    If some of the keys in the provided map are not variables
    in this expression, their provided value is simply ignored.
    If a value is provided for each variable in this linear
    combination, the resulting expression will not contain any
    variables of its own, only a constant.

    :   **Specified by:**: `plugInNumbers` in interface `MathExpr<V>` **Specified by:**: `plugInNumbers` in interface `Polynomial<V>`
    :   **Parameters:**: `numbers` - objects mapped to numerical values. **Returns:**: the linear combination obtained by "plugging in" the provided numerical values.

---


### plugIn

```
LinearComb<V> plugIn(java.util.Map<?,? extends LinearComb<? extends V>> linearCombs)
```

:   Plugs in the provided linear combinations to produce a new
    linear combination.

    If some of the keys in the provided map are not variables
    in this expression, their provided value is simply ignored.

    :   **Parameters:**: `linearCombs` - objects mapped to linear combinations. **Returns:**: the linear combination obtained by "plugging in" the provided linear combinations.

---


### plugIn

```
LinearComb<V> plugIn(java.util.Map<?,java.lang.Double> numbers,
                     java.util.Map<?,? extends LinearComb<? extends V>> linearCombs)
```

:   Plugs in the provided numerical values and linear combinations to
    produce a new linear combination.

    If some of the keys in either of the provided maps are not variables
    in this expression, their provided values are simply ignored.

    :   **Parameters:**: `numbers` - objects mapped to numerical values.: `linearCombs` - objects mapped to linear combinations. **Returns:**: the linear combination obtained by "plugging in" the provided numerical values and linear combinations. **See Also:**: `plugIn(Map)`, `plugInNumbers(Map)`

---


### getTermList

```
java.util.List<LinearComb.Term<V>> getTermList()
```

:   Returns the terms of this linear combination as list.

    :   **Returns:**: the terms of this linear combination as list.

---


### getTermArray

```
LinearComb.Term<V>[] getTermArray()
```

:   Returns the terms of this linear combination as an array.

    :   **Specified by:**: `getTermArray` in interface `Polynomial<V>`
    :   **Returns:**: the terms of this linear combination as an array.

---


### iterator

```
java.util.Iterator<LinearComb.Term<V>> iterator()
```

:   Returns an iterator over the terms of this linear combination.

    :   **Specified by:**: `iterator` in interface `java.lang.Iterable<LinearComb.Term<V>>`
    :   **Returns:**: an iterator over the terms of this linear combination.

---


### hashCode

```
int hashCode()
```

:   Returns a content-based hash code for this linear combination.

    :   **Specified by:**: `hashCode` in interface `MathExpr<V>` **Specified by:**: `hashCode` in interface `Polynomial<V>` **Overrides:**: `hashCode` in class `java.lang.Object`
    :   **Returns:**: a content-based hash code for this linear combination.

---


### equals

```
boolean equals(java.lang.Object obj)
```

:   Indicates if the provided object is equal to this
    linear combination.

    For two linear combinations to be equal, they must contain
    the same numerical constant and terms.

    :   **Specified by:**: `equals` in interface `MathExpr<V>` **Specified by:**: `equals` in interface `Polynomial<V>` **Overrides:**: `equals` in class `java.lang.Object`
    :   **Parameters:**: `obj` - the object to be tested for equality with this linear combination. **Returns:**: `true` if this linear combination equals the provided object; `false` otherwise. **See Also:**: `Polynomial.Term.equals(Object)`

---


### toString

```
java.lang.String toString()
```

:   Returns an appropriate string representation of this
    linear combination.

    :   **Specified by:**: `toString` in interface `MathExpr<V>` **Specified by:**: `toString` in interface `Polynomial<V>` **Overrides:**: `toString` in class `java.lang.Object`
    :   **Returns:**: an appropriate string representation of this linear combination.


---


|  |  |  |
| --- | --- | --- |
| **Overview** \| **Package** \| **Class** \| **Use** \| **Tree** \| **Deprecated** \| **Index** \| **Help** | |  |
| **PREV CLASS**   **NEXT CLASS** | **FRAMES**    **NO FRAMES**     **All Classes** |  |
| SUMMARY: NESTED \| FIELD \| CONSTR \| METHOD | DETAIL: FIELD \| CONSTR \| METHOD |  |


---
